# Unscheduled home consultations by registered nurses may reduce acute clinic visits

**DOI:** 10.1186/s12913-024-11643-3

**Published:** 2024-11-01

**Authors:** Karin Bergman, Lena Hedén, Annelie J Sundler, Malin Östman, Jenny Hallgren

**Affiliations:** 1 Faculty of Caring Science, Work Life and Social Welfare, University of Borås, Borås, Sweden; 2 Research, Education, Development & Innovation, Primary Health Care, Vänersborg, Region Västra Götaland Sweden; 3 General Practice / Family Medicine, School of Public Health and Community Medicine, Institute of Medicine, Sahlgrenska Academy, University of Gothenburg, Gothenburg, Sweden; 4 School of Health Sciences, University of Skövde, Skövde, Sweden

**Keywords:** Collaborative Health Care Model, Clinical decision-making, Nursing assessment, Referral, Consultation, Integrated care, Swedish health care direct, Ambulance services, Community home health care

## Abstract

**Background:**

To effectively utilize available healthcare resources, integrated care models are recommended. According to such model’s, registered nurses have the potential to increase patient access to health care services and alleviate organizational workload. Studies on acute home consultation assessments by registered nurses are sparse. The aim was to describe the reasons and actions for unscheduled same-day face-to-face registered nurse consultation at home offered to patients calling the national telephone helpline for healthcare in Sweden (SHD 1177), according to the integrated Collaborative Health Care model.

**Methods:**

A descriptive cross-sectional study was designed. Data from registered nurses (*n* = 259) working within the Collaborative Health Care model, who performed unscheduled consultations at home (*n* = 615) using a data collection tool from 2017 to 2018 were collected.

**Results:**

Among the 615 unscheduled home consultations performed by registered nurses, > 50% of the patients were managed at home as their health problems were not deemed as requiring a same-day referral to a clinic when assessed by the registered nurses. The most frequent health problems and reasons for contact were urinary tract problems, followed by medical and surgical conditions. Social factors, including living alone, impacted referral. Those living with a partner received care at home to a greater extent than those who lived alone.

**Conclusion:**

An integrated model for healthcare involving registered nurses direct assessment, action and accountability seems to be an efficient option for providing integrated care at home and reducing acute clinic visits.

**Supplementary Information:**

The online version contains supplementary material available at 10.1186/s12913-024-11643-3.

## Introduction

Organizing healthcare to meet the population’s needs is a global challenge [1]. Aging populations, increase in the prevalence of long-term health problems and difficulties recruiting and retaining professional labor in the healthcare system, are challenges that drive the need to shift and transfer healthcare from hospital-based to primary care and community-based services [2, 3]. Different forms of acute care models and healthcare services at-home, for adults and older adults have been developed to meet the needs for acute assessments and treatments when health deteriorates [4–6]. In addition, various efforts have been made to implement healthcare models for more integrated primary care and community-based services, in which registered nurses (RNs) manage and prioritize patient flows [7]. Homecare interventions are suggested to be cost-saving and effective alternatives to hospital care [5].

Currently, integrated care is the leading model for improving healthcare systems worldwide [1]. Integrated care aims to deliver seamless, coordinated, and comprehensive care across different healthcare settings and providers [8]. This involves collaboration among various healthcare professionals to ensure that patients receive comprehensive and holistic care that meets their needs [1]. There are different arrangements for integrated care services, including primary, secondary, and community home healthcare [9] and community paramedicine [10]. Although evidence for other outcomes, such as service costs, remains unclear, integrated care models are expected to improve patient satisfaction, increase perceived quality of care, and enable access to services [9]. Nevertheless, integrated care remains a complex phenomenon that occurs at multiple levels, with various interventions, stakeholders, and contextual factors influencing its processes and results [8]. However, few interventions have been evaluated regarding acute assessments and treatments when health deteriorates in the home environment; these interventions are aimed at relieving the organizational workload and pressure on primary care physicians or emergency departments.

Primary care is central to the healthcare system in most Western countries, where primary care is the first level of contact for individuals seeking care. In Swedish primary care, the patient’s first contact is commonly with an RN via telephone (telenursing) at a primary healthcare center or the national medical helpline - Swedish health care direct (SHD 1177), who conducts an initial assessment and prioritization of the patient’s problems and needs. However, telephone nursing assessments can be complex and challenging [11, 12]. The complexity of telenursing involves making an accurate and safe assessment of the care needs of unknown individuals via telephone without visual cues and sometimes by proxy via the individual’s relative [13]. Occasionally, an additional face-to-face assessment is required to make decisions regarding further actions. Research on RNs’ decision-making strategies in acute face-to-face home consultations are sparse [7].

It has been argued that RNs’ skill set is uniquely positioned to provide a significant contribution to the primary care context [14]. However, there is a lack of research to clarify the contributions regarding the unique role of RNs [15]. There is some evidence indicating that integrating RNs into primary care has the potential to increase patient access to a primary care provider [16], by manage patient flow, guiding telephone triage and prioritizing patient appointment access and service coordination [14]. It has been claimed that RNs can provide patient care that is comparable and complementary to that of other primary care providers, specifically with respect to patient satisfaction, enablement, self-reported quality of life, self-efficacy, and improvements in health behaviors’ [17]. There is a need for unscheduled same-day assessments in patients’ homes [10, 18]. Nonetheless, studies on unscheduled same-day home consultation assessments by RNs are lacking.

The Collaborative Health Care (CHC) is a locally developed integrated care model implemented in parts of Sweden to coordinate available resources based on patients’ needs, provide a coherent healthcare system, and prevent avoidable hospital admissions [19]. As a part of the CHC model, patients can be assessed and treated at home by RNs, as an alternative to same-day referral to a clinic when telephone assessment is deemed insufficient. To effectively utilize available healthcare resources, there is a need for more integrated primary care and community-based services.

### Aim

The present study aimed to describe the reasons and actions for unscheduled same-day face-to-face RN consultations at home offered to patients calling SHD 1177, according to the CHC model.

## Materials and methods

### Design

A descriptive cross-sectional study was designed. The STROBE checklist for reporting cross-sectional studies was used to improve reporting quality, enhance comparability and facilitate critical appraisal [20].

### Setting and sample

The CHC model is based on collaboration between primary care, hospital care, ambulance services, community home healthcare, and the SHD 1177. The CHC model was initiated, developed and tested in 2009 on a small scale in two municipalities. Since then, developed and gradually spread, and by 2023 the CHC model was temporarily implemented in a total of 15 municipalities in west Sweden. The current study included home consultation by a RN to patients with health problems needing same-day assessment, but not necessarily a physician consultation or clinic visit, who called the national telephone helpline for healthcare in Sweden. Criteria for offering home consultation were cases assessed by the RN at SHD 1177 as either health problems that could be managed with a home consultation by a RN, or when remote assessment via telephone was deemed insufficient and more information was needed e.g. vital signs and physical examination. As support for assessment of patients’ health problems, the RNs at SHD 1177 has a digital decision support system [21]. Ordinarily, these patients would have been referred by the SHD 1177 to a primary healthcare center or the emergency department but were now given an option with a home consultation by a RN to evaluate whether their problem could be managed at home as an alternative to referral. Regarding telephone consultation, the choice of whether to call and which physician is decided by the individual RN who made the home consultation visits.

### Participants and data collection

A convenience sample of 615 home consultations performed by RNs from ambulance services (*n* = 94), single-ambulance responders (*n* = 69), and community home healthcare services (*n* = 452) were collected during 2017–2018. The home consultations were performed in 13 home healthcare districts and eight ambulance/single responder districts in 13 municipalities in a region in West Sweden, covering a population of approximately 150.000 of the total 10.2 million inhabitants in Sweden. A data collection tool (supplementary material) with questions related to the home consultations was completed by the RNs who performed the unscheduled home consultations on the same day.

### Measurements

The data was collected using a data collection tool. The data collection tool included questions on (A) patient demographics, (B) date and time of home consultation, (C) reasons for home consultations, (D) actions performed by the RNs during the visit, (E) telephone consultation at home consultation, and (F) outcome of home consultations, that is, what level of care the patient continued to receive after the home consultation.

A) The demographic background variables included patient age, sex, social living status (living together or alone), type of housing (house/apartment), and location of residence (urban/rural).
B) The time distribution for home consultations refers to the time at which the home consultation assessment was carried out by the RN. The time variable was merged into six categories: (1) daytime during weekdays (07.00–16.59), (2) evening during weekdays (17.00–20.59/21.59), (3) nights (21.00/22.00–06.59) and three categories on weekends, Friday evening until Monday morning, (4) daytime (07.00–16.59), (5) evenings (17.00–20.59/21.59), and (6) nights (21.00/22.00–06.59).
C) Reasons for home consultation were classified into 10 categories: (1) medical conditions: respiratory problems and chest pain. allergies, diabetes, and infections; (2) surgical conditions: abdominal pain, constipation, nausea, vomiting, ulcers, and nosebleeds; (3) neurological conditions: headache, dizziness, seizures, unconsciousness, and neurological symptoms; (4) psychiatric conditions: confusion/delirium and anxiety; (5) orthopedic conditions: arm/leg/neck/back ailments and pain; (6) urinary tract problems, excluding tract infections; (7) fall accidents; (8) care coordination: care planning, coordination, support for patients and relatives, security alarms, and follow-up visits; (9) drug management/medical technology tasks: drug management, medical technological tasks, electrocardiogram tests and checks; and (10) other conditions: assessment of general condition, pain/aches, and nonspecific malaise.
D) The RNs’ actions refer to the types of care and treatments performed during the home consultation and were classified into 10 categories: (1) health assessment: assessment of health condition, care needs, and appropriate level of continued care; (2) information and patient education; (3) care coordination; (4) drug administration; (5) emotional support; (6) wound dressing; (7) urinary catheter; (8) blood and urine sample collection; (9) medical technology task (intravenous access, cannula, and sonde management); and (10) other corresponding measures that do not fit into existing categories, such as “bowel treatment,” “support bandage.” “compression treatment,” “plaster cast management,” “nosebleed treatment,” “on-site treatment,” and “others.“.
E) Telephone consultations with a physician regarding home consultations are cases in which the RN needs support from a physician in assessing the patient.
F) The outcome of home consultations refers to the level of care that the patient continued to receive after home consultation assessment by the RN. The outcomes of home consultation management by RNs include the following: “remains at home”(1) and “same-day referrals to clinics for physician consultation”(2).

### Data analysis

Descriptive statistics were used to describe demographic background variables, reason for the home consultation, RNs’ actions at home consultations, telephone consultation with a physician regarding the home consultations, and outcome of home consultations. The chi-square test was used to test associations between demographic background variables and the outcome of home consultations. To allow for comparisons of age groups and the outcome variable, dichotomous variables were created, in which each age group (1) was compared to the other patients (0), and the comparison was made for each age group to explore whether patients in any of the age groups benefited more or less from home consultations in terms of being able to remain at home instead of being referred for a clinic visit. All the data were analyzed using IBM SPSS Version 28.0. The significance level was set at α = 0.05 (5%).

### Ethical considerations

This study followed the guidelines of the Declaration of Helsinki and was approved by the Regional Research Ethics Board in Linkoping, Sweden (DNR: 2017/376 − 31). The participating RNs were informed about the content and aim of the study and that their participation was voluntary and that they could withdraw at any time. After completing the data collection tool, the RNs consented to participate.

## Results

**Fig. 1:**
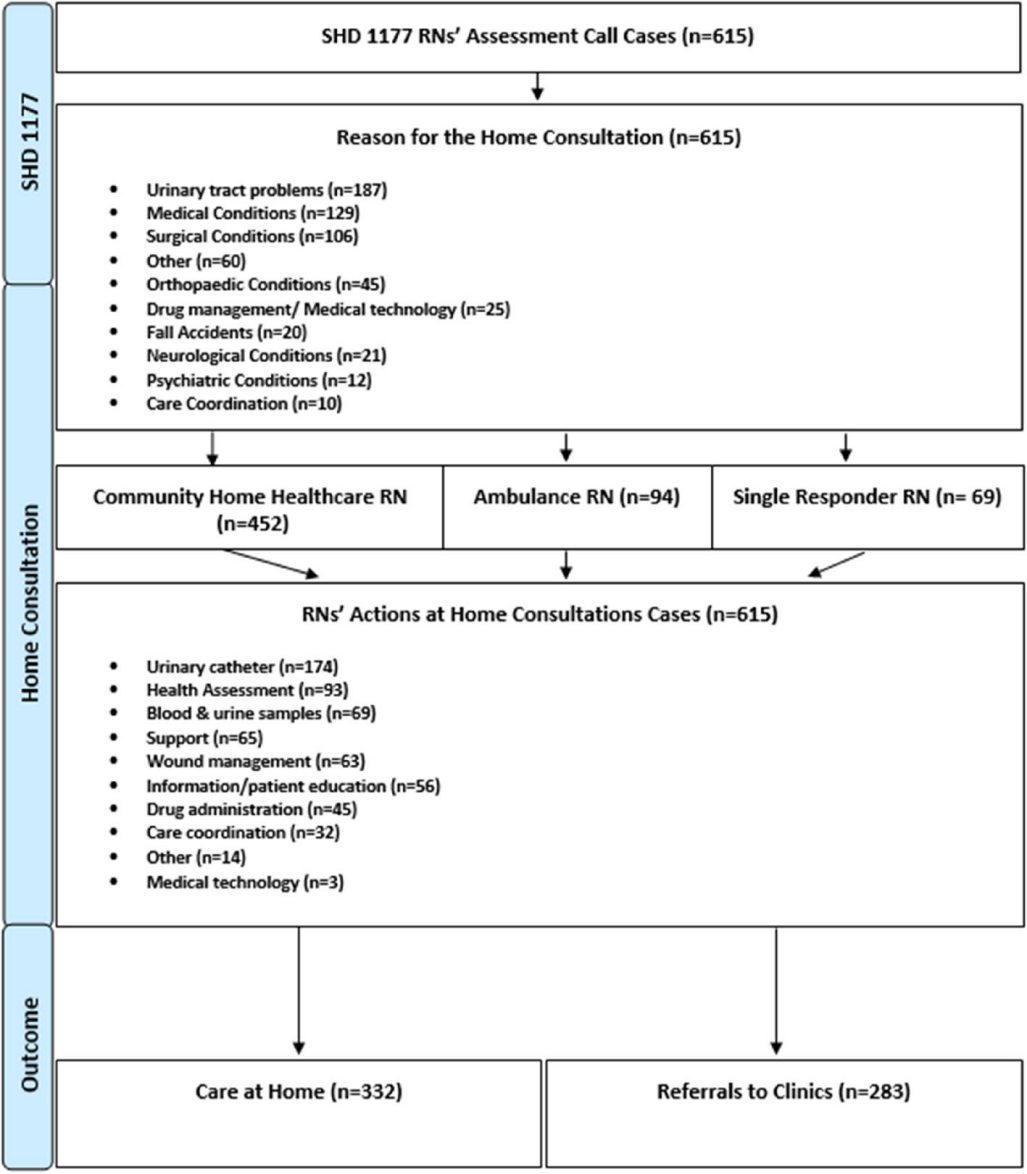
Overview of unscheduled home consultations, reasons for the consultations, actions performed by RNs during consultations, and outcome of home consultations

In total, 615 home consultations performed by RNs (*n* = 259) employed in community home healthcare services (*n* = 183) or ambulance services (*n* = 76) were included; for an overview of the included home consultations, see Fig. 1. Of the patients who received home consultations, most people were older and there was an even distribution of men and women, for further characteristics see Table 1.

**Table 1:** Characteristics of cases and outcome of home consultations

	Total %(*N*)
**Sex**	100 (615)
Male	52.4 (322)
Female	47.6 (293)
**Age group**	100 (606)
0–18	5.4 (33)
19–39	8.6 (52)
40–64	9.9 (60)
65–74	16.2 (98)
75–85	33.7 (204)
86-	26.2 (159)
**Social living status**	100 (436)
Living together	52.8 (230)
Living alone	47.2 (206)
**Type of Housing**	100 (433)
House	60.3 (261)
Apartment	39.7 (172)
**Residence**	100 (431)
Urban	61.9 (267)
Rural	38.1 (164)

### Home consultation time distribution

Most home consultations (88,0%, *n* = 483) were outperformed during out-of-office hours, and 12,0% (*n* = 66) were conducted during weekday office hours. Of the consultations, 48,1% (*n* = 264) were performed on weekends.

### Reasons for home consultations

The most frequently reported health problems were urinary tract problems (30.4%), followed by medical (21.1%) and surgical conditions (17.2%). Male patients most frequently reported on urinary tract problems (42.9%), whereas women most frequently reported medical conditions (25.3%), see Table 2.

**Table 2:** Reason for the home consultation and RNs’ actions and telephone consultation across sex and age group

	Total % *N* = 615	Sex %		Age group %					
		Male (*n* = 322)	Female (*n* = 293)	0–18 (*n* = 33)	19–39 (*n* = 52)	40–64 (*n* = 60)	65–74 (*n* = 98)	75–85 (*n* = 204)	86- (*n* = 159)
**Reason for the home consultation**									
Medical Conditions	21.1	17.1	25.3	78.8	13.5	25.0	17.3	15.7	18.9
Surgical Conditions	17.2	13.0	21.8	15.2	17.3	16.7	15.3	19.1	17.0
Neurological Conditions	3.4	3.1	3.8	0.0	9.6	6.7	3.1	3.9	0.6
Psychiatric Conditions	2.0	1.6	2.4	0.0	3.8	0.0	4.1	2.0	1.3
Orthopaedic Conditions	7.3	4.0	10.9	0.0	3.8	10.0	7.1	9.8	5.7
Urinary tract problems	30.4	42.9	16.7	0.0	38.5	21.7	30.6	29.9	37.1
Fall Accidents	3.3	3.1	3.4	0.0	1.9	1.7	2.0	4.9	3.8
Care Coordination	1.6	1.6	1.7	0.0	0.0	1.7	3.1	2.5	0.6
Drug management/ Medical technology	4.1	4.3	3.8	0.0	3.8	8.3	8.2	2.0	3.1
Other	9.8	9.3	10.3	6.1	7.7	8.3	9.2	10.3	11.9
**RNs’ actions at home consultation**									
Health Assessment	15.1	14.9	15.4	27.3	13.5	20.0	12.4	17.2	11.3
Information/patient education	9.1	7.5	11.0	18.2	9.6	16.7	5.2	7.4	8.8
Care coordination	5.2	2.5	8.2	9.1	9.6	5.0	4.1	5.4	3.8
Drug administration	7.3	6.8	7,9	24.2	5.8	8.3	6.2	5.4	5.7
Support	10.6	9.0	12.3	6.1	1.9	11.7	14.4	12.7	8.8
Wound management	10.3	8.1	12.7	3.0	7.7	11.7	14.4	10.8	8.8
Urinary catheter	28.3	41.6	13.7	0.0	44.2	11.7	25.8	29.4	35.2
Blood & urine samples	11.2	7,8	15.1	12.1	7.7	15.0	9.3	8.8	15.7
Medical technology	0.5	0.3	0.7	0.0	0.0	0.0	1.0	1.0	0.0
Other	2.3	1.6	3.1	0.0	0.0	0.0	7.2	2.0	1.9
**Telephone consultation at home consultation**									
No Telephone consultation	84.1	82.9	85.3	63.6	82.7	71.7	84.7	86.8	89.3
General Practitioner	7.8	8.4	7.2	6.1	5.8	10.0	9.2	8.8	6.3
Specialists at the Hospital	7.3	8.1	6.5	30.3	7.7	16.7	5.1	4.4	3.8
Other consultation	0.8	0.6	1.0	0.0	3.8	1.7	1.0	0.0	0.6

### RNs’ actions performed during home consultations

The most frequently reported action performed during home consultations by the RNs was urinary catheterization (28.3%), followed by health assessment (15.1%) and blood and urine sample collection (11.2%), as shown in Table 2.

### Health problems for which RNs need to consult another healthcare professional

During home consultations, RNs needed to consult another healthcare professional for their assessment in 16% of the patients. Among the patients aged 0–18 years, a specialist physician at the hospital was consulted in 30.3% of the patients. Among the oldest age category, ≥ 86 years, the RNs assessed patients independently in 89.3% of the patients and consulted specialist physicians at the hospital in 3.8% of the patients (Table 2).

### Outcome of home consultations

In most cases (54.0%), patients who were offered a same-day consultation at home by an RN required no further referrals to a clinic. However, 46.0% of the patients were referred to an acute consultation in primary care, a specialist clinic at the hospital, or an emergency department visit (Table 3). In patients who remained at home, we observed a greater proportion of male patients (53.9%) than female patients (46.1%), in contrast to patients referred to clinics (50.5% vs. 49.5%, respectively), although no significant difference was observed between the sexes.

**Table 3:** Characteristics of cases and outcome of home consultations

	Remains at home %(*n*)	Referrals to clinics %(*n*)	*P*-value
**Total**	54 (332)	46 (283)	
**Sex**			0.402
Male	53.9 (179)	50.5 (143)	
Female	46.1 (153)	49.5 (140)	
**Age group**			
0–18	6.5 (21)	4.3 (12)	0.253
19–39	7.7 (25)	9.6 (27)	0.372
40–64	11.1 (36)	8.5 (24)	0.325
65–74	13.5 (44)	19.2 (54)	0.049*
75–85	38.1 (124)	28.5 (80)	0.017*
86-	23.1 (75)	29.9 (84)	0.045*
**Social living status**			0.024*
Living together	57.9 (132)	47.1 (98)	
Living alone	42.1 (96)	52.9 (110)	
**Type of Housing**			0.941
House	60.4 (136)	60.1 (125)	
Apartment	39.6 (89)	39.9 (83)	
**Residence**			0.299
Urban	64.3 (144)	59.4 (123)	
Rural	35.7 (80)	40.6 (84)	

Contingency tables Chi-Squared Test (X²) α = 0.05 (5%) *P* < 0.05 significant result *

The age category of patients who most frequently remained at home after consultations was 75–85 years (38.1%). In contrast, patients who were referred to clinics with physician consultation were mostly aged ≥ 86 years (29.9%) (Table 3).

Significant associations between the outcome of home consultations were observed in three of the older age groups (Table 3) related to their social living status. Among patients who remained at home we observed a larger proportion of patients who lived with a partner than lived alone. Reversely, a larger proportion of patients living alone were referred to a clinic compared to those living together. The analysis indicated a significant association between the outcome of home consultations and social living status (*p* = 0.024*). Moreover, more patients living in urban areas (64.3%) than in rural areas (35.7%) received care at home, although this association was not significant (Table 3).

Based on reasons for home consultation actions performed by the RNs during the visit, the demand for home consultation by RNs consisted of a heterogeneous group of patients. Furthermore, a greater proportion of the consultations remained at home for urinary tract problems than for cases referred to clinics. The number of consultations via telephone to general practitioners was greater in patients who remained at home than in patients referred to clinics (8.4% vs. 7.%), whereas the number of consultations via telephone to specialists was greater in patients who were eventually referred for clinic visits than in patients who remained at home (6.6% vs. 8.1%) (Table 4).

**Table 4:** Outcome of home consultations in patients managed at home versus patients being referred

	Total %(*N*)	Remains at home %(*n*)	Referrals to clinics %(*n*)
**Reason for the home consultation**	100 (615)	100 (332)	100 (283)
Medical Conditions	21.0(129)	20.2 (67)	21.9 (62)
Surgical Conditions	17.2 (106)	16.6 (55)	18.0 (51)
Neurological Conditions	3.4 (21)	2.4 (8)	4.6 (13)
Psychiatric Conditions	2.0 (12)	1.5 (5)	2.5 (7)
Orthopaedic Conditions	7.3 (45)	6.9 (23)	7.8(22)
Urinary tract problems	30.4(187)	32.8 (109)	27.6 (78)
Fall Accidents	3.3 (20)	3.6 (12)	2.8 (8)
Care Coordination	1.6(10)	2.1 (7)	1.1 (3)
Drugs / Medical technology	4.1 (25)	4.5 (15)	3.5 (10)
Other	9.8 (60)	9.3 (31)	10.3 (29)
**RNs’ actions at home consultation**	100 (614)	100 (332)	100 (282)
Health Assessment	15.2 (93)	13.6 (45)	17.0 (48)
Information/patient education	9.1 (56)	11.1 (37)	6.7 (19)
Care coordination	5.2 (32)	4.2 (14)	6.4 (18)
Drug administration	7.3 (45)	8.4 (28)	6.0 (17)
Support	10.6 (65)	11.8 (39)	9.2 (26)
Wound management	10.3 (63)	8.4 (28)	12.4 (35)
Urinary catheter	28.3 (174)	29.2 (97)	27.3 (77)
Blood & urine samples	11.2 (69)	10.2 (34)	12.4 (35)
Medical technology	0.5 (3)	0.3 (1)	0.7 (2)
Other	2.3 (14)	2.7 (9)	1.8 (5)
**Telephone consultation at ** **home consultation**	100 (615)	100 (332)	100 (283)
No Telephone consultation	84.1 (517)	84.6 (281)	83.4 (236)
General Practitioner	7.8 (48)	8.4 (28)	7.1 (20)
Specialists at the Hospital	7.3 (45)	6.6 (22)	8.1 (23)
Other consultation	0.8 (5)	0.3 (1)	1.4 (4)

## Discussion

Home consultations by RNs, according to the CHC model, were found as an alternative to clinic referrals in 54% of the cases observed in this study. In ordinary healthcare delivery, without the CHC model option, it is likely that these patients had been referred to a primary care clinic or emergency department for further assessment as telephone advice was not considered sufficient. Moreover, in 84.1% of the home consultations, the RNs independently managed these without further consulting any physician by telephone. These results demonstrate the benefits of home consultations by RNs for patients with health conditions that required to be assessed on the same day, which could reduce the need for acute clinic visits. The results show that 8 of the 10 home consultations were performed during out-of-office hours, which further emphasizes the CHC model’s relieving effect on acute healthcare services. This indicates that the CHC model was predominantly used when the primary care centers were closed and there was limited access to general practitioners. This knowledge is valuable for the redesign of healthcare systems. The current result is in line with previous research [22], arguing that using RNs to their full extent of practice in primary care is a promising avenue for enhancing both patients’ experience of care and health service efficiency. Previous research shows that the CHC model is highly appreciated by the older population, since they do not have to visit a clinic for health problems that can be managed at home [19]. Frail older persons might also risk negative outcomes due to hospitalization [23].

Of the patients assessed at home, 46% were subsequently referred to a clinic. This result highlights the uncertainty and complexity of remote telephone health assessments [11, 24]. It can be argued that if remote assessments were easy and straightforward, patients in need of physician consultations would have been referred directly to the clinic from their initial telephone contact with SHD 1177. Hence, same-day home consultation by the RNs would not have been deemed necessary in the first place. This notion is supported by the study in pre-hospital context, which showed that approximately 50% of ambulance-missions were assessed as non-urgent and claimed that the difference between a telephone-based assessment and direct observations was substantial [25]. The findings support that remote consultations are not always sufficient and that there are advantages with face-to-face assessments with additional access to nonverbal communication, vital signs, and physical examination [26].

The most frequently observed health problems and reasons for contact were urinary tract problems (30.4%), followed by medical (21.1%) and surgical conditions (17.2%). Notably, the frequency of urinary tract problems was high, accounting for almost one-third of the reasons for contact. Lower urinary tract symptoms are common in men, with 62% of men reporting at least one [27]. Furthermore, the incidence of Lower urinary tract symptom is increasing globally, increasing with age, and has a negative impact on quality of life [28]. The current results showed that RNs could handle 58% of urinary tract problems via home consultations, supporting the results of a study by Choi et al. [29], which indicated that community-based nurse-led care can effectively alleviate Lower urinary tract symptom and enhance self-efficacy.

A significant association was observed between age group and telephone consultation with a physician. A greater proportion of RNs consulted a physician for children aged 0–18 years (36.3%) than for those aged ≥ 86 years (10.0%). This might indicate that child assessment was challenging for RNs, which could be related to limited training regarding pediatrics in undergraduate RN education in Sweden [30]. RNs in community home healthcare services commonly care for older adults and thus have more clinical experience in geriatrics than pediatric competence [31].

A significant difference in the outcome of home consultations and social living status was observed; a greater proportion of those living together remained at home than those who lived alone. This result highlights the importance of family caregivers and social support, which are often prerequisites for continuing patient care at home [32]. These findings indicate that RNs consider a patient’s social situation when performing assessments. Hence, their decisions may not only consider medical data and severity. This result is in line with Holmberg’s suggestion that decisions should not only be based on medical severity but also be guided by information on patients’ living conditions and social support [33].

### Strengths and limitations

An authentic observation sample in a real-life clinical situation can be regarded as a strength that reduces the risk of speculation and response bias. A sample size of 615 patients was considered sufficiently large for this purpose. A limitation of the study is that no a priori sample size calculation was performed prior to data collection, and the design is not intended to perform analysis on causal relationships. Thus, we could only present descriptions of demand for unscheduled RNs home consultations and which patients benefited from the consultation as an alternative to a referral to a clinic.

The study limitations also included a lack of information regarding the participating RNs’ age, specialist education status, and length of work experience. Owing to the nonrandom convenience sample used and differences in RN education and healthcare organizations, the results may not be generalizable to other regions or internationally.

Independent health assessment by RNs of patients seeking primary care for acute conditions is a relatively new and growing area within the RN’s scope of practice. More research is needed on communication, assessment, decision making, and priorities in RNs’ consultations and knowledge that can be implemented in the education and training of RNs to ensure healthcare service quality and patient safety.

### Conclusion

This study indicates that home consultations by RNs could be an option to manage patients’ needs and conditions at home, to avoid acute clinic visits. Frail older persons could benefit the most from such services, as these individuals might risk negative outcomes due to hospitalisation. An integrated model for healthcare involving RNs’ direct assessment, action and accountability may be an efficient option for providing integrated care at home and reducing avoidable acute clinic visits.

## Supplementary Information

Supplementary Material 1.

## Data Availability

No datasets were generated or analysed during the current study.

## Abbreviations

CHC: Collaborative Health Care
RN: Registered nurse
SHD 1177: The national medical helpline - Swedish healthcare direct
