# Supplementary material for: Unscheduled home consultations by registered nurses may reduce acute clinic visits
Source: BMC Health Serv Res. 2024 Nov 1;24:1338. doi: 10.1186/s12913-024-11643-3 (PMC11531198; doi:10.1186/s12913-024-11643-3)
Supplement: Supplementary file 1 — Supplementary Material 1. [file 12913_2024_11643_MOESM1_ESM.docx]

**Here you register assignments that you have received within Collaborative Health Care Model**

**Date**

| 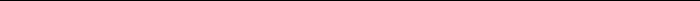 |
| --- |

**Time**

| 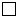 | 07.00-09.00 |
| --- | --- |
| 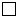 | 09.00-11.00 |
| 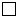 | 11.00- 13.00 |
| 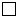 | 13.00-15.00 |
| 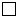 | 15.00-17.00 |
| 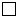 | 17.00-19.00 |
| 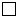 | 19.00-21.00 |
| 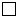 | 21.00-23.00 |
| 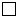 | 23.00-01.00 |
| 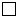 | 01.00-03.00 |
| 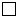 | 03.00-05.00 |
| 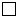 | 05.00-07.00 |

**Where did you get the assignment from?**

| 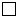 | Swedish Healthcare Direct, SHD 1177 |
| --- | --- |
| 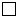 | SOS Alarm |
| 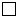 | Ambulance Services |
| 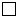 | Municipal Home Healthcare |
| 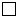 | Primary Health Care Centre |
| 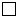 | Hospital department of Geriatric Medicine |
| 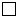 | Municipal Home Social Service |

**State from which health centre you received the assignment**

| 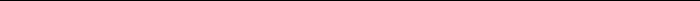 |
| --- |

**What kind of mission was it?**

| 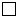 | SHD 1177-mission |
| --- | --- |
| 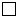 | While Waiting for Ambulance (WWFA) |
| 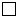 | Assistance-mission |

**Did you go on the mission?**

| 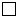 | Yes | |
| --- | --- | --- |
| 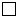 | Yes, but the mission was aborted | |
| 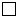 | No, because the ambulance was deemed to arrive earlier | |
| 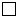 | No, due to assignments in own business | |
| Comment: | | |
| 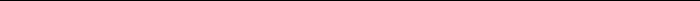 | | |

**What was the reason for the home visit?**

| 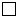 | Asssistans to aid mobility, lift |
| --- | --- |
| 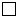 | ECG |
| 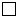 | Follow-up visit |
| 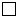 | Breathing problems |
| 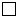 | Heart and lung problems - chest pains |
| 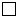 | Heart and lung problems - cardiac arrest |
| 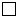 | Infectious disorders - fever |
| 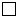 | Infectious disorders - wound infection |
| 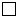 | Infectious disorders - urinary tract infection |
| 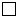 | Surgical problems - burn |
| 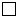 | Surgical problems - abdominal pain |
| 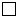 | Surgical problems - constipation |
| 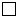 | Surgical problems - vomiting, nausea |
| 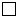 | Surgical problems - urinary tract problems (except UTI) |
| 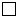 | Surgical complications - wound damage |
| 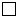 | Neurological problems - withdrawal symptoms |
| 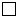 | Neurological problems - headache |
| 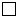 | Neurological problems - convulsions |
| 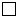 | Neurological disorders - unconsciousness |
| 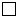 | Orthopedic problems - arm / hand or leg / foot problems |
| 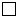 | Orthopedic problems - neck problems |
| 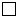 | Orthopedic problems - back problems |
| 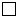 | Psychiatric disorders - confusion |
| 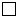 | Psychiatric disorders - worry / anxiety |
| 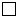 | Other - allergy |
| 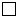 | Other - family support |
| 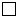 | Other - diabetes |
| 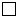 | Other – fall accidents |
| 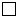 | Other - drug management |
| 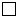 | Other - epistaxis |
| 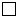 | Other - security alarm |
| 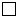 | Other: |
|  | 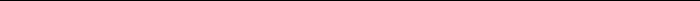 |

**Actions at home**

| 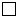 | CPR |
| --- | --- |
| 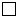 | Information / Self-care |
| 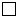 | Drug administration |
| 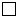 | Coordination - between patient and other care provider - make an appointment at primary care centre |
| 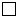 | Assessment |
| 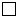 | Placement of drainage / tube / cannula |
| 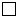 | Insertion of a i.v. catheter |
| 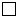 | Sampling / tests |
| 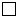 | Wound dressing |
| 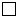 | Bowel treatment |
| 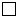 | Urinary catheter |
| 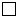 | Emotional social & practical support |
| 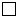 | Other |
|  | 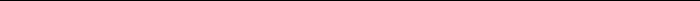 |

**Did the patient agree to transfer information to another healthcare provider?**

| 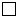 | Yes |
| --- | --- |
| 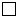 | No |

**Patient age:**

| 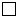 | 0-18 yr |
| --- | --- |
| 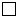 | 19-39 yr |
| 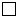 | 40-64 yr |
| 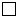 | 65-74 yr |
| 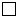 | 75-85 yr |
| 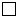 | 86 - |

**Patient sex:**

| 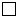 | Male |
| --- | --- |
| 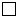 | Female |

**Does the patient live alone or live together?**

| 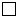 | live alone |
| --- | --- |
| 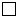 | live together |

**Does the patient live in a rural or urban area?**

| 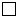 | Rural |
| --- | --- |
| 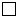 | Urban |

**The patient lives in type of housing:**

| 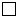 | House |
| --- | --- |
| 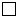 | Apartment |
| 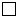 | Municipal care home |

**Is the patient enrolled in municipal health care?**

| 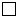 | yes |
| --- | --- |
| 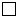 | No |
| 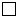 | Do not know |

**Did you contact a physician for consultation during the assignment?**

| 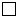 | Yes, at the primary health centre |
| --- | --- |
| 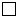 | Yes, at the SOS Alarm emergency services |
| 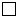 | Yes, at the emergency department |
| 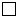 | Yes, a physician in the helicopter |
|  | No |
|  | Other |
|  |  |

**The patient's care level outcome:**

|  | Emergency department |
| --- | --- |
|  | Hospital department of Geriatric Medicine |
|  | Emergency department other location |
|  | Self care at home |
|  | Booked appointment at primary care centre |
|  | Emergency visit at Out of office primary health care centre |
|  | Emergency visit at primary Health care centre |
|  | Other |
|  |  |

**Mode of transport:**

|  | No transportation |
| --- | --- |
|  | Ambulance |
|  | Non urgent Medical transport |
|  | Single responder |
|  | Own transport |
|  | Do not know |
